# Supplementary material for: Patterns of Intron Gain and Loss in Fungi
Source: PLoS Biol. 2004 Nov 30;2(12):e422. doi: 10.1371/journal.pbio.0020422 (PMC532390; doi:10.1371/journal.pbio.0020422)
Supplement: Table S1 — Also available at http://genes.mit.edu/NielsenEtAl/. (4.3 MB ZIP). [file pbio.0020422.st001.zip › NielsenEtAl/html/1294.html]

AN2980.1.NCU09109.1.MG09927.1.FG01278.1


```
 CLUSTAL W (1.82) Multiple Sequence Alignments - Introns Inserted


Sequence 1: MG09927.1	105 aa
Sequence 2: FG01278.1	109 aa
Sequence 3: NCU09109.1	91 aa
Sequence 4: AN2980.1	109 aa
Alignment Length: 109 aa
Number Identitical Residues: 62 aa
Alignment Score (without introns) 3046


MG09927.1 	MPTEAGHRL1YVK2GRHLSYQRSRHTNRPETS~LIKIEGVDDTNAAK~Y0----KVAYVY
NCU09109.1	MPTEAGHRL1YVN2------------------~LIKIEGVDDTAAAN2F~YLGKRVAYVY
FG01278.1 	MPSEAGHRL1YVK2GRHLSYQRSRHTTRPATS~LIKIEGVDDTNAAN2F~YLGKKVAFVY
AN2980.1  	MPSEHGHRL1YVK2GRHLSYQRSKRAVNPNTS2LIKIDGVDNTEAAN2F~YLGKKVAFVY
          	**:* **** **: .   : . :  :  . :: ****:***:* **: :   ..:**:**

MG09927.1 	RAQKEVRGTKIRVIWGKVTRPHG1NSGVVRAKFTTPLPTRSFGASVRIMLYPSSI
NCU09109.1	RGQKEVRGTKIRVIWGKVTRPHG1NSGVVRAKFAAPLPSRSFGASVRIMLYPSSI
FG01278.1 	RAQKEIRGTKIRVIWGKVTRPHG1NSGVVRAKFTSPLPTKSFGASVRVMLYPSSI
AN2980.1  	RAKREVRGSNIRVIWGKVTRPHG1NSGVVRAQFRHNLPAKSFGATVRVMLYPSNI
          	*.::*:**::************* *******:*   **::****:**:*****.*
```
